# Vegetable substrates as an alternative for the inclusion of lactic acid bacteria with probiotic potential in food matrices

**DOI:** 10.1007/s13197-023-05779-z

**Published:** 2023-07-16

**Authors:** Camila Bernal-Castro, Elpidia Espinosa-Poveda, Carolina Gutiérrez-Cortés, Consuelo Díaz-Moreno

**Affiliations:** 1 Facultad de Ciencias, Doctorado en Biotecnología, Universidad Nacional de Colombia, Bogotá, Colombia; 2 Departamento de Nutrición Humana, Facultad de Medicina, Universidad Nacional de Colombia, Bogotá, Colombia; 3 Universidad Nacional Abierta y a Distancia (UNAD), Escuela de Ciencias Agrícolas, Pecuarias y del Medio Ambiente (ECAPMA), Bogotá, Colombia; 4 Instituto de Ciencia y Tecnología de Alimentos (ICTA), Universidad Nacional de Colombia, Bogotá, Colombia

**Keywords:** Gut microbiota, Prebiotics, Bioprocess, Food matrices, Bioactive compounds

## Abstract

Vegetable substrates are food matrices with micronutrients, antioxidants, and fiber content with a high potential for bioprocesses development. In addition, they have been recognized as essential sources of a wide range of phytochemicals that, individually or in combination, can act as bioactive compounds with potential benefits to health due to their antioxidant and antimicrobial activity and recently due to their status as prebiotics in the balance of the human intestinal microbiota. This systematic review explores the benefits of lactic fermentation of plant matrices such as fruits, vegetables, legumes, and cereals by bacteria with probiotic potential, guaranteeing cell viability (10^6^–10^7^ CFU/mL) and generating bioactive metabolic products for modulation of the gut microbiome.

## Introduction

The term “microbiota” refers to the population of microorganisms in a given location (Ballan et al. 2020). Although microorganisms colonize every area of the human body, most are found in the gastrointestinal tract. The intestinal microbiota encompasses more than 1000 species of bacteria and is the group of microorganisms with the most incredible abundance of those belonging to the phyla of Bacteroidetes and Firmicutes (Novik and Savich 2020). Lactic acid bacteria (LAB) are the main microorganisms used for probiotic purposes. They are an essential part of the human and mammalian gut microbiota, especially for exerting significant health-promoting effects in the host (Li et al. 2020). For example, viable and metabolic active LAB found in food products such as fermented milk, meat, fruits, and vegetables has been used as functional foods to benefit from their advantageous properties, such as producing metabolites with bio-therapeutic interest (Erginkaya and Konuray-Altun 2022).

The ability to manipulate this community of microorganisms has been recognized for decades through the consumption of probiotics (live microorganisms), prebiotics (selective substrates for their growth), and the combination of these two, known as symbiotics (Hojsak et al. 2018). This has determined that probiotics with diverse bacterial communities have metabolic, barrier, and trophic functions. Consequently, intestinal microbiota composition characterization is relevant in microbiology, immunology, and food biotechnology. Therefore there is a new interest in new substrates that allow the viability of probiotic bacteria and the production of metabolites of interest (Gu and Roberts 2019). In the industry, the inclusion of microorganisms with probiotic potential has traditionally been carried out in dairy derivatives (cheese, yogurt, ice cream, among others) and supplements in the form of tablets, capsules, and lyophilized preparations (Bernal Castro et al. 2017).

The most commonly used medium for obtaining probiotic bacteria biomass is Man, Rogosa, and Sharpe (MRS). However, some studies have suggested that these reactive grade substrates could reduce functionality due to excluding the potential synergistic effect of bioactive compounds such as fibers and antioxidants (Vinderola et al. 2019). Furthermore, in terms of dairy products, specific sectors of the population, such as those allergic to milk proteins, lactose intolerant, and strictly vegetarians, cannot consume these matrices; therefore, the need has arisen to offer consumers an alternative to fermented dairy products by exploring new non-dairy matrices as probiotic-inclusion vehicles (Valero-Cases et al. 2020).

Including probiotics in substrates of vegetables is an innovative field within food biotechnology. Several challenges are related to selecting the specific strains to be used, the inoculum production, and the bacteria's viability during storage under cooling conditions (4 °C). In addition, the plant substrate’s intrinsic characteristics include pH, acidity, and the composition of phytonutrients and micronutrients on the viability of bacteria with probiotic potential (Flach et al. 2017). The period of survival and residence of probiotics in the colon can be influenced by the dose and the matrix used as a substrate for the probiotics (Valero-Cases et al. 2020). This article aims to review the lactic fermentation of substrates of vegetable origin (fruits, vegetables, legumes, cereals, and vegetables) by including lactic acid bacteria with probiotic potential and its impact on the balance of the human intestinal microbiota.

## Literature search methodology

A careful literature search was conducted to identify experimental studies or literature reviews addressing the lactic fermentation of vegetable substrates by probiotics as a strategy to balance the human gut microbiota. The literature review was carried out in the following databases searched: Science Direct, Medline via PubMed, Scopus, and Taylor and Francis. The inclusion criteria for selecting articles were (a) Articles published in English from 2010 to 2021, with specific prevalence in articles with less than 5 years of publication to date; (b) original articles and reviews related to the probiotic's inclusion in vegetable substrates and the interaction with the human gut microbiota. Articles not related to the topic were excluded; (c) Review articles on the metabolism of bioactive compounds in lactic fermentation bioprocess were included in the primary search for the information. They were included in their keywords or sections on probiotics and prebiotics; (d) Chapters from books about the incidence of the metabolism of lactic acid bacteria with probiotic potential in vegetable matrices.

## Metabolism of lactic acid bacteria (LAB) with probiotic potential

The microorganisms most used as probiotics belong to the lactic acid bacteria (LAB) group, the most common genera being *Lactobacillus* sp., *Enterococcus* sp., and *Bifidobacterium* (Yahfoufi et al. 2018). These bacterial groups produce substances with high industrial applicability, such as bacteriocins, vitamins, and exopolysaccharides. Lactic acid is the main metabolic product of LAB, which this heterogeneous group is named (Novik and Savich 2020). They can be divided into two groups according to their metabolism: homofermentative or heterofermentative. Homofermentative cultures produce two lactic acid molecules from one glucose molecule through the Embden–Meyerhof–Parnas's pathway (glycolysis). Heterofermentative bacteria can be compulsory or facultative. The latter catabolizes glucose into ethanol, CO_2_, and lactic acid. Mandatory heterofermentative LAB prefers the 6-phosphogluconate/phosphoketolase pathway, while facultative ones use both biochemical pathways (Oliveira et al. 2018). *Bifidobacterium* is saccharolytic and uses host-derived oligosaccharides for growth and survival. These microorganisms use a particular pathway called a bifid shunt, resulting in lactate and acetate production. This metabolic pathway is more effective in generating energy adenosine triphosphate (ATP) production through hexoses' catabolism than glycolysis (Luo et al. 2020).

Initially, the term “probiotic” was used by Lilly and Stillwell in 1965 as a counterpart to the term antibiotic, “A microbial substance capable of stimulating the growth of other microorganisms” (Ross and Preedy 2016). However, in 2014 an expert consensus document by the International Scientific Association for Probiotics and Prebiotics (ISAPP) defined probiotics as “live microorganisms that confer a host health benefit in adequate amounts” (Hill et al. 2014). Recently, a new approach to the probiotics concept has been gaining attention due probiotics are a colony of bacteria that live in our intestines and are regarded as a metabolic ‘organ’ due to their beneficial effects on human health, including metabolism and immunological function (Tegegne and Kebede 2022).

The possible mechanisms of action of probiotics include improvement of the epithelial barrier function (Cremon et al. 2018), modification of the intestinal microbiota (Novik and Savich 2020) (Novik and Savich 2020), optimization of the immune system (Gu and Roberts 2019), competition with pathogenic bacteria for mucosal adherence (Chatterjee et al. 2017), antimicrobial effects (O’Bryan et al. 2014) and anti-inflammatory (Aguilar-Toalá et al. 2018).

Probiotic formulations include a single strain, multiple organisms, or a prebiotic (symbiotic) (Jana et al. 2020). Survival and stability of LABs with probiotic potential on different substrates are highly dependent on the strains, the physicochemical and bioactive composition of the culture medium, fermentation, and storage conditions (Min et al. 2018). For example, a low pH range of 2.5–3.7 represents a challenge in developing bioprocesses with probiotics. Since most of these bacteria are sensitive to such low pH conditions, in these circumstances, the working strain’s choice is essential since bacteria are required that can withstand highly acidic conditions and have acceptable viability (Bansal et al. 2016). The high adaptation of these bacteria to a wide diversity of substrates, such as milk, meat, fruits, vegetables, and cereals, significantly reduces their metabolic capacities. These bacteria depend on exogenous sources for amino acids, nucleic acid precursors, and vitamins, which are classified as demanding growth (Sauer et al. 2017). This disadvantage limits industrial production because the components of the culture media required for viability are expensive, and the purification of the products from the medium is generally more difficult (Bajpai 2020). These microorganisms prefer glucose and can metabolize several common hexoses. However, the ability to ferment other sugars depends on the strain. For example, some LAB in dairy substrates can use the most abundant sugar in milk, lactose, and carbon. In contrast, bacteria associated with non-dairy substrates use various other carbohydrates, including β-glucosides (Kowalczyk et al. 2015).

## Balance of the human gut microbiota

The gastrointestinal tract of humans and mammals, in general, has developed together with a diversity of microorganisms in a symbiotic relationship over millions of years; this microbial community is known as the intestinal microbiota (Wu et al. 2021). Factors such as intestinal morphology, pH variations in different intestines, oxygen, and the type of nutrients available can explain the host’s specific intestinal microbiota. The gut microbiota comprises different bacteria taxonomically classified by genus, family, order, and species, with other metabolic functions.

However, some phyla predominate in the composition: Bacteroidetes, Firmicutes, Actinobacteria, Proteobacteria, Verrucomicrobia, Fusobacteria, a limited number of Archaea, mainly methanogens (Wan et al. 2021). Throughout life, the more diverse this microbial community is, the better it will resist external threats. The gut microbiota represents a changing population that can be modified by intrinsic factors of the host (genetics, age) and extrinsic factors (body mass index, smoking, physical activity) and mainly by diet. However, it is recommended that there is a balance between the host and the microorganisms in the gastrointestinal tract for metabolic and immune functions and to prevent diseases (Rinninella et al. 2019) (Table 1). The LAB performs essential physiological processes, such as producing vitamin K and biotin, regulating intestinal motility, transforming bile acids and steroids, metabolizing indigestible fibers, such as β-glucans, absorbing minerals, and regulating toxins and mutagens (Novik and Savich 2020). In addition, some microorganisms produce short-chain fatty acids (SCFAs) that provide energy to the colonic mucosa and peripheral body tissues and influence water absorption present in the colon and fecal pH (Luo et al. 2020).

**Table 1:** Metabolic function of the phyla and representative genera of the human gut microbiota

Phyla	Example	Metabolic function	References
*Firmicutes*	*Clostridium Lactobacillus*	It is one of the predominant phyla that colonize the intestinal tract of mammals, representing 90% of the total intestinal bacteria They include several species identified as dominant butyrate producers and specialized no digestible polysaccharide degrader	Cao et al. (2019)
*Bacteroidetes*	*Bacteroides,* *Prevotella*	It accounts for about 30% of all gut bacteria, and it is more common in western populations consuming a high-fat or high-sucrose diet They are commensal inhabitants of the gastrointestinal tract resistant to bile by the action of the hydrolase of bile salts	Danneskiold-Samsøe et al. (2018)
*Proteobacteria*	*Escherichia* *Klebsiella o*	Microorganisms that present indicates dysbiosis with less presence in the healthy microbiota Potential pathogenic microorganisms	Rajoka et al. (2017)
*Actinobacteria*	*Bifidobacterium,* *Collinsella*	Microorganisms with probiotic potential due to the production of short chain fatty acids (SCFA) They can induce weight loss by decreasing intestinal permeability	Shortt et al. (2018)
*Fusobacteria*	*Fucobacteriaceae*	They produce various organic acids, such as acetic, propionic, butyric, formic or succinic acid, depending on the substrate	Graf et al. (2015)
*Verrucomicrobia*	*Akkermansia*	They represent 1% to 4% of the fecal microbiota of healthy people These bacteria break down the mucin that resides in the mucus layer	Roy (2017)
Archaea	*Methanobrevibacter*	They remove the final H_2_ product from bacterial fermentation, facilitating the fermentation rate and colonic energy production in the form of SCFA	Graf et al. (2015)

These metabolites improve the intestinal barrier, inhibiting the colonization of pathogens and the production of toxic elements, and they are used by intestinal cells as colonocytes to grow. Therefore, consuming plant-based beverages fermented with probiotics enriches the gut microbiota population and improves glucose metabolism (Valero-Cases et al. 2020).

The imbalance in the intestine’s bacterial composition, known as dysbiosis, is associated with a series of diseases related to inflammatory factors, such as obesity, inflammatory bowel disease, diabetes, and cancer (Ballan et al. 2020). Also, both commensal and pathogenic microbiota have complex interactions with their hosts. Understanding these interactions may show how these microbes alter human signalling pathways and lead to new therapeutic strategies, including designing new plant-based fermented products (Zhang et al. 2023).

Gut homeostasis is maintained through optimal interactions between the host and commensal microorganisms by communicating through the gut-associated lymphoid tissues (GALT) (Yahfoufi et al. 2018). Two lines of defense protect against dysbiosis: the mechanical and immune barriers. The automatic wall comprises polarized intestinal epithelial cells, enterocytes, and mucus lined in a single layer. The immune barrier consists of Peyer’s patches, mesenteric lymph nodes, immunoglobulin A (IgA), lymphocytes, macrophages, neutrophils, and natural killer (NK) cells that are found primarily within the lamina propria of the intestinal mucosa (Zhang et al. 2015).

However, various studies also reported that one of the essential cytoprotective effects of probiotics in the gut mucosa is strengthening the tight epithelial junctions and preserving mucosal barrier function. Probiotics enhance barrier function by inducing synthesis and assembly of tight junction proteins and prevent disruption of tight junctions by injurious factors (Krishna Rao and Samak 2013; Jana et al. 2020).

Through various mechanisms, bioactive compounds present in food can positively affect the intestinal microbiota’s composition or activity, thus restoring homeostasis in the intestine (dubious). Some of these food components have been described by the term “eubiotics,” dietary fiber, prebiotics, and secondary plant metabolites such as polyphenols and isoflavones are included within eubiotics (Wan et al. 2021). Phenolic compounds and dietary fiber have been analyzation separately and considered unrelated, probably due to differences in their physicochemical, biological, and metabolic pathways. However, it has been suggested that they may follow a standard and synergistic physiological process within the gastrointestinal tract (Sauceda et al. 2017). The consumption of probiotics and phytochemicals is becoming a possible therapeutic alternative for various syndromes and metabolic disorders, such as irritable bowel, even diabetes, and, recently, depression (Plaza-Diaz et al. 2019).

## Interaction between phytochemicals and the human intestinal microbiota

A possible mechanism in the microbiota modulation is epigenetics, i.e., genetic changes in genetic activity not attributable to modifications in the DNA sequence. Nutritional epigenetics study how healthy and bioactive compounds influence genetic activity (DNA methylation, histone modification, or micro RNAs' positive/negative regulation). These alterations increase or limit protein activity, changing molecular pathways in the host. For example, fiber, polyphenols, and carotenoids are essential in maintaining homeostasis by influencing epigenetic mechanisms (Graham et al. 2017). In addition, vegetable substrates are considered sources of molecules with bioactivity that impact human health and interact with the intestinal microbiota (Septembre-Malaterre et al. 2018).

Phytochemicals are non-nutritive bioactive compounds in vegetables, fruits, tea, grains, and nuts. These compounds have specific therapeutic potential and include carotenoids, phenols, fibers, alkaloids, flavonoids, tannins, terpenoids, glycosides, saponins, and anthraquinones (Koley et al. 2019).

**Fig. 1:**
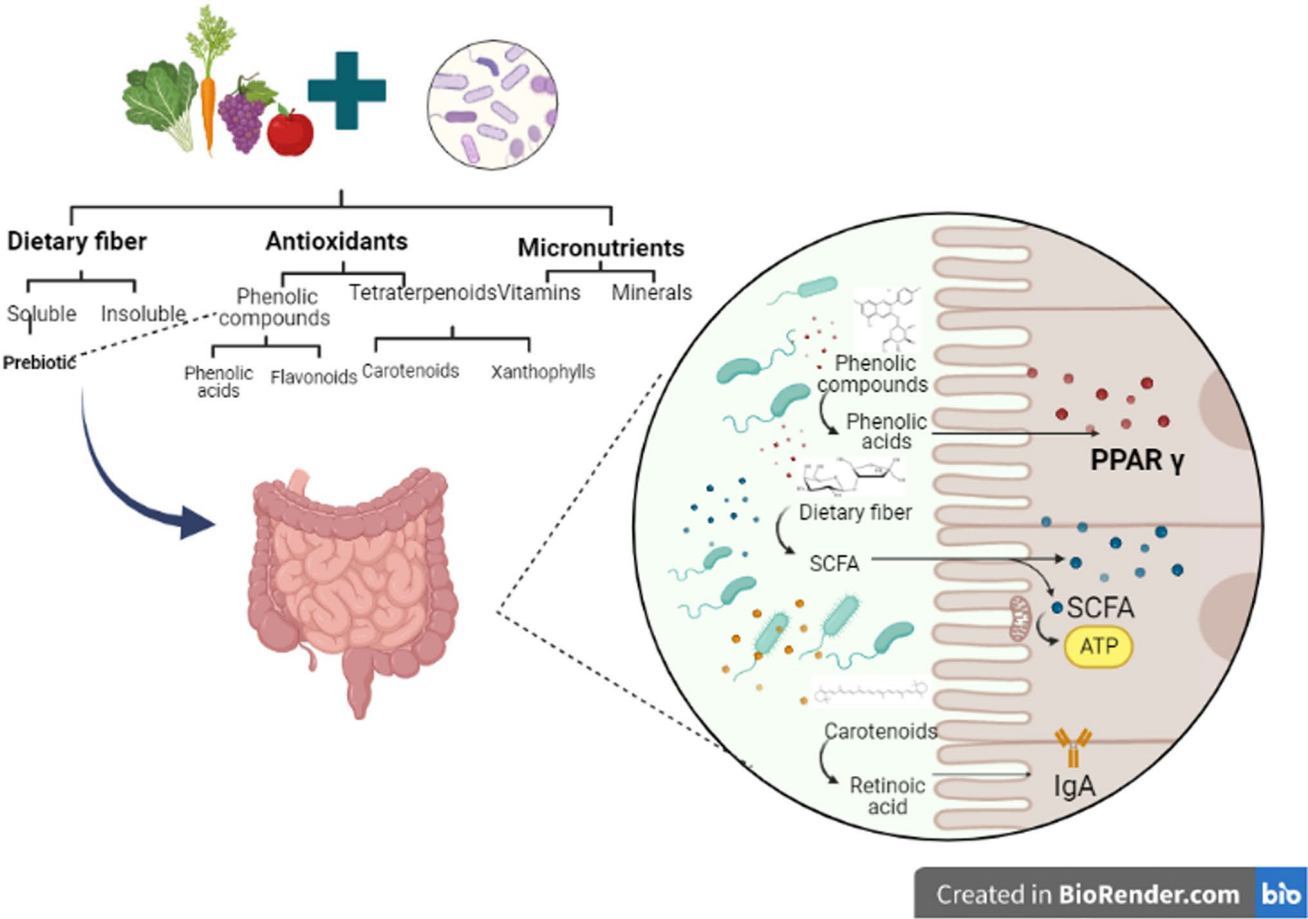
Interaction between phytochemicals and probiotics on the human gut microbiota. Source: (Tegegne and Kebede 2022; Liu et al. 2023; Zhang et al. 2023)

As described in Fig. 1, biologically plausible reasons for the protective association of phytochemicals are their antioxidant and antimicrobial activity (Yahia et al. 2019). In addition, numerous studies revealed that phytochemicals entering the intestinal system could beneficially alter the composition of microbial ecology by acting as prebiotics and antimicrobial agents against pathogenic gut microbiota. Therefore, further investigations should focus on examining the therapeutic potential of phytochemicals and explaining the specific influence of each bioactive compound in modulating the gut microbiota (Santhiravel et al. 2022).

Nevertheless, the molecular mechanisms and their interactions with intestinal microorganisms remain poorly understood (Yin et al. 2019). *Nevertheless, *in vitro and in vivo experiments have shown that the phytochemicals could reduce the appearance of dysbiosis, the damage to the intestinal barrier, and bacterial translocation and can protect against gastrointestinal problems through various biochemical reactions and physiological processes (Martínez et al. 2020).

There is a growing trend to use compounds of plant origin to treat or prevent oxidative stress due to the antioxidant properties of phytochemicals, their low toxicity, high compatibility with the body, and increased clinical efficiency against free radicals (Martínez et al. 2020). An antioxidant is a molecule that slows the oxidation process or neutralizes the free radical in lower concentrations, reducing the damaging effect.

Antioxidants can protect cells through a variety of mechanisms. Such as the conversion of reactive oxygen species (ROS) to no radical species, the removal of pro-oxidative metal ions, and a decrease in molecular oxygen concentrations, intake of exogenous antioxidants, such as ascorbic acid, α-tocopherol, carotenoids, and polyphenols, present in fruits, vegetables, beverages, cereals, and other food products (Yin et al. 2019).

Another functional characteristic of phytochemicals is their antimicrobial activity due to the presence of polyphenolic compounds. The antimicrobial mechanisms of action of phytochemicals are related to changes in cell morphology, alteration of the membrane associated with loss of ions, reduction of membrane potential, changes in the proteome and transcriptome, inhibition of ATP-activity, suppression of virulence factors, inhibition of nucleic acid synthesis, alteration of the composition of the lipid profile, mitochondrial dysfunction, and deterioration in the use of carbon sources (Prakash et al. 2020). The antimicrobial and antioxidant properties of phytochemicals, as well as the ability to modulate the composition of the gut microbiota, have extended the concept of prebiotics to “a substrate that is selectively used by host microorganisms that confer a health benefit” (Gibson et al. 2017) that is not limited only to fermentable carbohydrates.

## Inclusion of probiotics and prebiotics in non-dairy substrates

The understanding that phytochemicals act as prebiotics in the intestinal lumen may account for some controversial observations regarding these molecules, including their limited bioavailability, highly varied structures, multiple targets of action, diverse bioactivity, and high-dose toxicity (Martel et al. 2020). LAB-mediated fermentations using vegetable substrates are bioprocesses that allow the production of a high proportion of bioactive substances such as short-chain fatty acids (SCFAs), phenolic acids, and bioactive peptides, among others. The LAB strains isolated from spontaneous fermentations of vegetable matrices have shown to be promising probiotic candidates. Recently a select group of LAB known as neutrophilic lactic acid bacteria (FLAB) has been described. This category is defined as bacterial strains that prefer fructose to glucose as a growth substrate (Cagno et al. 2015).

According to Table 2, the variety of vegetable matrices used as vehicles for the inclusion of bioactive, probiotic, and prebiotic compounds in the human gastrointestinal tract can be observed, among which are the following:

- **Fermented fruits and vegetables:** the adaptation of LABs to fruits and vegetables is markedly varied because of the diversity of the ecological niches present in these matrices and the microbial capacity to share metabolic energy between biosynthesis and cell maintenance (global stress responses). A wide variety of research has focused on the production of substrates with symbiotic characteristics, including various types of mixed-type vegetables or fruits, for example, carrot (*Daucus carota*) and orange (*Citrus X Sinensis*) with different concentrations of inulin (Valero-Cases and Frutos 2017).
- **Fermented legumes:** legumes are potential probiotic vehicles due to their indigestible oligosaccharides content; legumes' carbohydrate profiles are fructooligosaccharides (FOS), resistant starch, and sugar alcohols that can serve as prebiotics. Within legumes, the most widely used is soybeans (*Glycine max*, L.) due to their high-quality protein and mineral content, and isoflavones, which can reduce the incidence of osteoporosis and symptoms of menopause (Valero-Cases and Frutos 2017).
- **Fermented cereals:** cereals can be used to design fermented substrates with probiotic characteristics because they contain nutrients easily assimilated, like cell wall polysaccharides (β-glucan), fructans, and arabinogalactan Within cereals, oats (*Avena sativa*, L.) are a potential functional ingredient, due to their proteins, soluble fiber, and antioxidant properties, with β-glucan being the most important fraction of carbohydrates due to its prebiotic properties in the intestine (Angelov et al. 2018).These studies show that lactic acid fermentation mediated by probiotic bacteria can increase bacterial cells' viability and improve the food matrix's functional aspects. Some of these aspects are mentioned below:
- **Viability of probiotic cultures:** the viability of the probiotics is strain and specific plant matrix, which has led several studies to select suitable probiotics for each substrate (Bansal et al. 2016) (see Table 2). Probiotic strains can be successfully incorporated into plant matrices where they can retain their viability and produce bioactive substances, extend shelf life, and positively impact human health. However, the composition of a substrate (i.e., fat content, protein type, carbohydrates, and pH) can affect probiotics’ growth and survival (Neffe-skoci et al. 2018). Probiotic functionality differs based on dose, the composition of the food matrix, and strain; understanding the probiotics’ genus and species is necessary to accomplish the desired effects on the host (Tegegne and Kebede 2022). The main factors that affect the viability and activity of probiotic cultures include pH, titratable acidity, water activity, incubation temperature, presence of salt, sugar, and chemicals, such as hydrogen peroxide, molecular oxygen, bacteriocins, artificial flavors, coloring agents, heat treatment, inoculation rate, and proportion, strain species, packaging materials, and conditions, storage methods and conditions (Aspri et al. 2020). The stomach acidity, bile salts, enzymes such as lysozyme present in the intestine, toxic metabolites, including phenols produced during digestion, bacteriophages, antibiotics, and anaerobic conditions could also affect the probiotic´s viability
- **Increased antioxidant activity:** Fermentation can improve plant substrates' antioxidant activity by increasing the release of flavonoids. Fermentation-induced structural degradation of cereal cell walls can also release and induce the synthesis of various bioactive compounds through phenolic conversion and depolymerization of high molecular weight phenolic compounds (Hur et al. 2014). The mechanisms of antioxidant action of LAB with probiotic potential are not fully known. Among the possible mechanisms is the ability to modulate the redox state of the human host through the chelation of metal ions, antioxidant systems, signaling pathways, and the production of microbial enzymes, such as glucosidase, amylase, cellulase, chitinase, inulinase, phytase, and xylanase (Wang et al. 2017).
- **Hydrolysis of phenolic compounds:** in recent decades, there has been a growing interest in the bioconversion of polyphenols by the intestinal microbiota. *Lactobacillus* species have received significant attention due to their high population in the gastrointestinal tract and their metabolic versatility. Several LAB species have demonstrated their ability to deglycosylate, de esterify, decarboxylate, and demethylate polymeric phenolic compounds into simpler compounds (phenolic acids) (Ryu et al. 2019), with β-glucosidase being one of the main enzymes responsible for the hydrolysis of flavonoids during lactic fermentation. In addition, it has been reported that while some phenolic compounds have an inhibitory effect (salicylic acid, methyl gallate, caffeic acid, ferulic acid, p-coumaric acid), others, such as gallic acid, free anthocyanins, catechin, and quercetin seem to activate the growth of some LAB (*Oenococcus oeni*).

**Table 2:** Studies on the interaction between plant substrates and their impact on the viability of probiotic bacteria

Vegetable matrix	Substrate characteristics	Inoculation and viability	Final substrate composition	Reference
Mango, açaí, banana and passion fruit	**Substratum:** Mixture of açaí pulp (AS) and banana (80:20 v/v) Mango (MS) and passion fruit mixture (60:40 v/v) **Initial pH:** 3.8 (AS) and 4.63 (MS)	**Strain:** *Lactobacillus acidophilus* LA-3 **Inoculum:** 9 log CFU/mL **Storage time**: 28 days 4 °C **Final viability:** 7.5 and 8.5 log CFU/mL in AS and MS respectively	**Sugars:** decrease in glucose, fructose and maltose (*p* < 0.05) **Organic acids:** higher content of lactic and succinic acids (*p* < 0.05) **Total phenols**: increased phenolic content (*p* < 0.05) **Final pH:** 3.60 (AS) and 4.25 (MS)	Alencar et al. (2018)
Mango	**Substratum:** Mango in liquid medium with enzymatic treatment (Trizyme P50 pectinases 0.8% and pH 5.0) and pasteurization **Initial pH:** 4.5	**Strain:** *Lactobacillus acidophilus* (MTCC10307), *Lactobacillus delbrueckii* (MTCC911), *Lactobacillus plantarum* (MTCC9511) and **Lactobacillus casei** **Inoculum: > **5 log CFU/mL (5%) **Storage time:** 30 days 4 °C **Final viability:** 8.0 log CFU/mL	**Sugars**: glucose reduction from 12% (p/v) to 5.8% (w/v) **Final pH:** 3.2	Reddy et al. (2015)
Mango and sapote	**Substratum:** Mango and sapote in liquid medium with enzymatic treatment (commercial pectinase 0.1% w/v) and pasteurization **Initial pH:** 4.2	**Strain:** *Lactobacillus plantarum* NCDC LP 20 **Inoculum**: 5 CFU/mL CFU/mL **Storage time:** 30 days 4 °C **Final viability** was no determinate	**Total phenols:** higher content of total phenols in sapote (145 mg/mL) compared to mango (60.24 mg/mL) **Final pH**: 3.2	Kumar et al. (2015)
Mango and Açaí	**Substratum:** Mix of mango and açaí pulps (7: 3% w/v) Heat treatment: ultra-pasteurization **Initial pH:** 4.55	**Strain:** *Lactobacillus rhamnosus* GG (Culturelle®) **Inoculum:** 10 log CFU/mL **Storage time:** 30 days 4 °C **Final viability:** greater than 7 log CFU/mL	**Total phenols**: 7221.96 to 7115.35 mg gallic acid/100 g Anthocyanins: 848.61 to 777.33 mg/100 g **Final pH**: 4.08	Moreira et al. (2017)
Orange	**Substrate: o**range, acid ascorbic and oligofructose **Total soluble solids (SST):** 8.22 to 10.77° Brix **Titratable acidity:** 0.53 to 0.63% citric acid **Initial pH:** 3.88 to 4.11	**Strain:** *Lactobacillus paracasei* ssp. *paracasei* (*L. casei*-01, Christian Hansen®) **Inoculum:** 8.0 CFU/mL **Storage time**: 28 days 4 °C **Final viability**: greater than 7 log CFU/mL	**Oligo fructose:** 1.65–1.8 g/100 mL **Titratable acidity:** 0.58 to 0.65% citric acid **Total soluble solids (TSS):** 8.22 to 10.77° Brix **Ascorbic acid:** 35.90 to 47.8 mg/100 g	da Costa et al. (2017)
Blueberry grenade Lemon	**Substrate:** Liquid blueberry medium: water (83%), concentrated cranberry (27%), sugar (11 g/100 mL) and vitamin C (32 mg/100 mL). Pomegranate liquid medium: water (68%), pomegranate from concentrate (32%), sugar (11.7 g/100 mL), Lemon Lime Liquid Medium is made up of fresh lemon and lime (100%) **Heat treatment:** sterilization	**Strain:** *Lactobacillus plantarum* NCIMB 8826 **Inoculum:** 8.0 log CFU/mL **Storage time:** 42 days 4 °C **Final viability:** 2–4 log CFU/mL	**Final pH:** blueberry (pH 2.7), pomegranate (pH 3.5) and lemon and lime juices (pH 2.8)	Srisukchayakul et al. (2018)
Cherry	**Substrate:** Cherry concentrate was diluted with distilled water sterilized at 8° Brix **Heat treatment:** pasteurization **Initial pH:** in one group of samples, the pH was adjusted to 3.5 (using industrial sodium bicarbonate), while, in another group, the pH was kept intact (~ 2.6)	**Strain:** *Lactobacillus rhamnosus,* *Lactobacillus plantarum*** and** *Lactobacillus casei* TD4 **Inoculum:** 8.0 log CFU/mL **Storage time:** 28 days 4 °C **Final viability:** 8.0 log CFU/mL	**Total phenols:** Decrease from 201.6 to 189.5 mg gallic acid/100 mL (*p* < 0.05) Anthocyanins: 80.45 to 77.56 mg of cyanidin 3-glucoside/100 mL (*p* < 0.05)	Nematollahi et al. (2016)
Cherry Pineapple Carrot Tomato	**Substrate:** Cherry fruit and pineapple and vegetable carrot and tomato media hydrolysed from wheat flour, whey and MRS broth were used as controls **Treatment:** membrane sterilization	**Strain:** *L. plantarum* CIL6, *L. plantarum* 1MR2, *L. plantarum* C2, *L. plantarum* POM1, *L. plantarum* DC400 and *L. plantarum* CC3M8 **Inoculum:** 8.0 log CFU/ mL **Storage time:** 21 days 4 °C **Final viability**: decrease in viability of 0.8 log CFU/mL	**Sugars and organic acids:** regardless of the medium used, the stoichiometric relationship between glucose, fructose, sucrose, maltose, lactose, galactose and/or malic acid consumed	Filannino et al. (2014)
Synthetic fruit medium with antioxidants	**Substrate:** Sucrose, sodium citrate, citric acid powder, and distilled water supplemented with white grape seed extract, green tea extract, vitamin B2, vitamin B3, vitamin B6, vitamin C, and vitamin E **Treatment:** pasteurization	**Strain: HOWARU®** *Lactobacillus rhamnosus* HN001, HOWARU® *Bifidobacterium lactis* HN001 *and Lactobacillus paracasei* LPC 37 **Inoculum:** 8.32 log CFU/mL **Storage time:** 42 days 4 °C **Final viability:** 4.29 to 7.41 log CFU/mL,	**Antioxidants:** ingredients such as green tea extract, vitamin C and grape seed extract, which contain levels of antioxidants and have oxygen scavenging properties, can promote a more favourable environment for probiotic bacteria	Shah et al. (2010)
Pumpkin	**Substrate:** Pumpkin pulp and peel supplements (70–30 p/p) for the design of culture media **Treatment:** vacuum drying	**Strain:** *L. casei* **Inoculum:** 9 log CFU/mL **Storage time**: 35 days 8 and 32 °C **Final viability**: > 8 log CFU/g	**Protein:** higher protein concentration, 12.2 g/100 g in the probiotic media	Genevois et al. (2019)
Mango	**Substratum:** Liquid mango medium supplemented with 5% FOS Initial pH: 4.07 SST: 10° Brix **Heat treatment:** sterilization	**Strain:** *L. casei, L paracasei L* *rhamnosus* HN001 **Inoculum: 7**.1 log CFU/mL **Storage time:** 20 days 4 °C **Final viability**: > 6 log CFU/mL	**Sugars:** there were no significant changes in the concentration of glucose, fructose and sucrose (*p* > 0.05) **Final pH**: 3.8	Acevedo-Martínez et al. (2018)
Blackberry, strawberry, papaya	**Substrate:** Mix of strawberry (20% w/v), blackberry (10% w/v) and papaya (5%) supplemented with 1% inulin) **Initial pH:** 3.36 **SST**: 10° Brix **Heat treatment:** sterilization	**Strain:** *L. casei* subsp. *rhamnosus* **Inoculum:** 9.96 log CFU/mL **Storage time:** 28 days 4 °C **Final viability:** > 6 log CFU/mL	**Sugars:** there were no significant changes in the concentration of glucose, fructose and sucrose (p > 0.05) **Lactic acid:** There was no production of lactic acid **final pH**: 3.1	Bernal Castro et al. (2019)
Carambola (star fruit)	**Substrate:** Carambola liquid medium **Initial pH:** 4.41 **SST:** 7.09° Brix **Treatment:** membrane sterilization	**Strain**: *Lactobacillus helveticus* L10, *Lactobacillus paracasei* L26, *Lactobacillus rhamnosus* HN001 **Inoculum:** 7 log CFU/mL **Storage time**: 8 days 4 °C **Final viability**: > 8 log CFU/mL	**Lactic acid** The carambola with *L. rhamnosus* produced the highest amount of lactic acid	Lu et al. (2018)
Oats	**Substrate Oats:** Oats flakes 25% (w/w) oat flakes **Initial pH:** 6.45	**Strain:** *L. plantarum* LP09 **Inoculum:** log 7 CFU mL **Storage time:** 30 days 4 °C **Final viability:** 6.5 log CFU/mL	**Antioxidant activity:** increased availability of polyphenols and antioxidant activity (25 and 70% more, respectively) **Hydrolysis index** decreased the hydrolysis index in vitro	Luana et al. (2014)
Legumes	**Substrate**: soy, adzuki and mung bean preparations Legume sprouts pH: 4	**Strain:** *L. plantarum* 299v **Inoculum:** 8.00 log10 CFU/g Fermentation: 25 °C for 4 days **Viability** was not determined	**Antioxidant activity:** Sprouts rich in probiotics showed higher polyphenol oxidase activity	Złotek et al. (2019)

However, the commercial need for plant-based probiotics, the feasibility, and the development of adaptive fermentation biotechnologies for their products do not go hand in hand compared to dairy probiotics, at least for now (Vijaya Kumar et al. 2015). Therefore, the investigation of the synergism between the physical and bioactive composition of plant substrates and the functionality of native and commercial probiotic strains is of importance in the field of food biotechnology.

## Incidence of the metabolism of lactic acid bacteria with probiotic potential in vegetable matrices

The use of vegetables as substrates are increasing in the market, as they are considered a promising alternative in the design of nutraceuticals (Paramithiotis 2017). As mentioned above, LAB converts the carbohydrate content into organic acids (mainly lactic acid), which lowers the pH to around 4.0 or less, guaranteeing the plant matrices’ stability. A lower pH value restricts pathogenic bacteria’s growth (for example, *Escherichia coli, Salmonella spp.,* and *Staphylococcus aureus*) (Montet et al. 2014). The effect of the metabolism of probiotic bacteria present in plant substrates during lactic fermentation is described below:

- **Catabolism of carbohydrates:** homofermentative LAB (e.g., *Lactobacillus*) ferment hexoses through glycolysis reactions and the enzyme lactate dehydrogenase action, generating lactic acid as a product. However, in situations of slow growth and low rates of glycolytic flux, homofermentative bacteria change to mixed lactic acid fermentation with acetate, ethanol, and lactate as products (Paramithiotis 2017). Heterofermentative LAB (e.g., *Leuconostoc, Oenococcus*, and *Lactobacillus*) produce lactic acid plus an appreciable amount of ethanol, acetate, and CO_2_ through the 6-phosphogluconate/phosphokinase pathway (Montet et al. 2014).
- **Citrate metabolism:** besides sugars, several species of LAB can metabolize citrate. Citrate fermentation leads to the formation of volatile compounds (diacetyl, acetoin, and butanediol). In homofermentative LAB, citrate use protects against the stress created by acidic conditions. In heterofermentative LAB strains, an extra mole of ATP is generated per mole of citrate (Kowalczyk et al. 2015).
- **Fermentation of dietary fiber and production of short-chain fatty acids (SCFAs):** dietary fiber refers to the components of the cell wall of vegetables, fruits, and cereals; generally, polysaccharides, lignin, and oligosaccharides are not hydrolyzed by endogenous enzymes in the small intestine of humans and can be fermented by probiotics and commensal microorganisms (Floch 2018). In terms of chemical structure, there is no structural similarity between the different components of dietary fiber other than they are substances that, due to their chemical or physical nature, are not digested by human enzymes. Human enzymes do not digest dietary fiber and, therefore, it reaches the colon, where it constitutes the primary source of energy for probiotic and commensal bacteria of the human intestinal microbiota, whose fermentative activity leads to the generation of organic acids (acid lactic acid, succinic acid) and SCFAs (acetate, propionate, and butyrate) (Cremon et al. 2018).
- **Carotenoid metabolism**: the effect of lactic fermentation on carotenoid levels depends on the carotenoid involved, the matrix, and the fermentation conditions. Although specialized treatments can reduce carotenoid levels in a food matrix, they can sometimes be accompanied by increases in potential bioavailability (Mapelli-Brahm et al. 2020). In addition, it has been proposed that carotenoids, such as β-carotene, can contribute to intestinal homeostasis by directly regulating the production of immunoglobulin IgA and thus preventing and delaying the development of dysbiosis (Lyu et al. 2018).
- **Increased content of vitamins**: a higher content of vitamins has been reported during the lactic fermentation of plant substrates (Paramithiotis 2017),particularly vitamins of groups B and K. The availability of micronutrients is increased in probiotic fermented plant substrates due to the significant reduction of the phytase enzyme (Montet et al. 2014).
- **Catabolism of amino acids**: LAB can obtain energy from amino acids under conditions of limited nutrients and a pH control mechanism (microbial adaptation) (Cagno et al. 2015). It has been reported that the concentration of amino acids of a branched chain decreased during the fermentation of vegetable substrates (fruits and vegetables) by *Lactiplantibacillus plantarum* which were converted into their respective 2-ketoacids (Di Cagno et al. 2020).
- **Production of exopolysaccharides (EPS):** LAB could biosynthesis of exopolysaccharides from cereal sources (Kowalczyk et al. 2015). EPS has a variety of different structures, sizes, and sugar compositions and is classified into two groups: homopolysaccharides (HoPS), which consist of a type of monosaccharide (α-d-glucan, β-d-glucan, fructan, or a polyglactin); and heteropolysaccharides (HePS), which consist of different types of monosaccharides (d-glucose, d-galactose, l-rhamnose, and its derivatives) (Kowalczyk et al. 2015) Produced EPS by LAB with probiotic potential has been reported to enhance gastrointestinal (GI) colonization of harmless bacteria in the human gastrointestinal tract and play an essential role as prebiotics. Their unique and complex chemical structures make them valuable in the food industries. They have also been reported as antitumor substitutes, immunostimulators, immunomodulators, and antioxidant agents in the pharmaceutical industries (Adesulu-dahunsi et al. 2018). The in situ synthesis of EPS by LAB during milk fermentation improves the fermented product’s viscosity and texture, especially in shaken yogurts, since these biopolymers act as thickeners and emulsifiers. Additionally, these EPS can be used as fat substitutes in low-calorie products (i.e., low-fat mozzarella cheese) (Hebert et al. 2015).

Developing plant-based probiotic carriers is essential in promoting healthier alternatives to dairy-based substrates. A thorough grasp of the range of plant fermentable phytochemicals and the critical quality indicators of fermented products aids in producing high-quality functional bioprocess. The challenges in maintaining the high viability of probiotics in these types of fermentations as well as the physicochemical parameters, must be carefully controlled to guarantee functional properties (Valero-Cases et al. 2020; Mojikon et al. 2022).

## Conclusion

Studies with various plant substrates have demonstrated their feasibility by presenting probiotic concentrations above the recommended minimum (> 7 (Log CFU/mL). There is a bidirectional relationship between the metabolism of LAB with probiotic potential and the products derived from the lactic fermentation of vegetables (organic acids, short-chain fatty acids, free amino acids, and phenols, among others) to produce a change in the conformation of groups. These substrates represent potential carriers for probiotics, prebiotics, and bioactive compounds, being a biotechnological alternative in developing bioprocesses in the current nutritional and pharmaceutical areas. However, further research should be complemented by a comprehensive study of the parameters of the fermentation process, the confirmation through in vitro and in vivo studies of the proposed health claims for fermented plant substrates, including selected native microorganisms, and the bioprospecting of new strains with probiotic potential isolated from plant matrices and the generation of postbiotics.

## Data Availability

The data that support the findings of this study are available from the corresponding author, [Consuelo Díaz-Moreno], upon reasonable request.
